# Facial Motion Capture System Based on Facial Electromyogram and Electrooculogram for Immersive Social Virtual Reality Applications

**DOI:** 10.3390/s23073580

**Published:** 2023-03-29

**Authors:** Chunghwan Kim, Ho-Seung Cha, Junghwan Kim, HwyKuen Kwak, WooJin Lee, Chang-Hwan Im

**Affiliations:** 1 Department of Electronic Engineering, Hanyang University, Seoul 04763, Republic of Korea; chunghwk0466@hanyang.ac.kr (C.K.); hoseungcha@gmail.com (H.-S.C.); hwankid@hanyang.ac.kr (J.K.); 2 Hanwha Systems Co., Ltd., Seongnam 13524, Republic of Korea; hk79.kwak@hanwha.com; 3 Korea Research Institute for defense Technology Planning and Advancement, Jinju 52851, Republic of Korea; 7pray@krit.re.kr; 4 Department of Biomedical Engineering, Hanyang University, Seoul 04763, Republic of Korea

**Keywords:** virtual reality (VR), social network service (SNS), facial expression, electromyogram (EMG), electrooculogram (EOG)

## Abstract

With the rapid development of virtual reality (VR) technology and the market growth of social network services (SNS), VR-based SNS have been actively developed, in which 3D avatars interact with each other on behalf of the users. To provide the users with more immersive experiences in a metaverse, facial recognition technologies that can reproduce the user’s facial gestures on their personal avatar are required. However, it is generally difficult to employ traditional camera-based facial tracking technology to recognize the facial expressions of VR users because a large portion of the user’s face is occluded by a VR head-mounted display (HMD). To address this issue, attempts have been made to recognize users’ facial expressions based on facial electromyogram (fEMG) recorded around the eyes. fEMG-based facial expression recognition (FER) technology requires only tiny electrodes that can be readily embedded in the HMD pad that is in contact with the user’s facial skin. Additionally, electrodes recording fEMG signals can simultaneously acquire electrooculogram (EOG) signals, which can be used to track the user’s eyeball movements and detect eye blinks. In this study, we implemented an fEMG- and EOG-based FER system using ten electrodes arranged around the eyes, assuming a commercial VR HMD device. Our FER system could continuously capture various facial motions, including five different lip motions and two different eyebrow motions, from fEMG signals. Unlike previous fEMG-based FER systems that simply classified discrete expressions, with the proposed FER system, natural facial expressions could be continuously projected on the 3D avatar face using machine-learning-based regression with a new concept named the virtual blend shape weight, making it unnecessary to simultaneously record fEMG and camera images for each user. An EOG-based eye tracking system was also implemented for the detection of eye blinks and eye gaze directions using the same electrodes. These two technologies were simultaneously employed to implement a real-time facial motion capture system, which could successfully replicate the user’s facial expressions on a realistic avatar face in real time. To the best of our knowledge, the concurrent use of fEMG and EOG for facial motion capture has not been reported before.

## 1. Introduction

Recently, virtual reality (VR) technologies have been actively incorporated into social network services (SNS), leading to a new entertainment service called VR-based SNS, where virtual avatars interact with each other on behalf of the users [1]. Indeed, several VR-based SNSs such as VRChat and Facebook Space have been already launched [2,3]. In addition, the application of VR environments with virtual avatars has been rapidly adopted by researchers in other areas such as the rehabilitation of autism patients, social skills training for children, and cognitive training for the elderly [4,5,6]. In the VR-based SNSs using 3D avatars, facial expression recognition (FER) technologies that replicate natural facial expressions and gestures on personal avatar faces are important for allowing VR users to feel as though they are interacting with a real person [7,8,9]. There are several technologies that can project the user’s face on their virtual avatar face; however, most of them have been implemented with camera-based facial motion capture techniques [5]. Although camera-based FER enables high-quality real-time FER, it is generally difficult to apply this technique to VR-based SNSs because VR head-mounted display (HMD) devices cover a large part of the user’s face, especially around the eyes, which can hinder the camera’s ability to capture the user’s facial expressions.

Several attempts have been made to overcome the issue mentioned above, such as the use of interior infrared cameras [10,11] and electromyography (EMG) [12,13]. A small infrared camera with a short focus range can be placed inside the VR HMD to capture eyeball movements and eye gestures when the user wears the HMD. However, the short-focused infrared cameras are generally more expensive than commercial VR HMD devices are. Additionally, as two short-focused infrared cameras are typically placed inside the VR HMD, an additional external camera needs to be attached to the outside of the VR HMD to capture lip motions. For example, Justus et al. developed a facial motion capture system using interior infrared cameras and an additional external camera for tracking facial expressions in covered and uncovered facial parts, respectively [14]; however, in their paper, the authors also mentioned that the use of two types of cameras increased the overall price and weight of the VR HMD.

EMG signals, which are biological electric signals generated by muscular activity, can provide an alternative way to address the issues of the conventional camera-based FER in VR environments. Since facial gestures are generated by combinations of various facial muscle movements, it is possible to predict facial gestures by analyzing facial EMG (fEMG) [13]. fEMG-based FER methods require only a few surface electrodes to capture fEMG signals, which can be readily implemented with a low-cost biosignal recording unit (e.g., TI ADS1299 chipset). In particular, electrodes can be easily embedded in the pad of commercial VR HMDs.

However, although there have been a series of fEMG-based FER studies [15,16,17,18,19], except for one by Cha et al. [15,16], the electrode locations of all studies were not determined considering the VR HMD environment. Cha et al. [19] developed an fEMG-based FER system that could successfully classify 11 facial expressions in real time using eight electrodes attached around the eyes, assuming the use of a commercial VR HMD. However, all the previous FER systems only classified discrete facial expressions, and thus, continuous changes in facial expressions could not be predicted and directly projected onto the 3D virtual avatar face in real time. Because the implementation of more realistic avatar expressions can provide the VR-based SNS users with a more immersive experience, the development of a new FER system that can predict continuous changes in facial expressions is necessary.

In this study, we designed a machine-learning-based FER system that can predict not only the types of the user’s facial expressions, but also the intensities of the muscle movements, and project the continuous facial expressions on to the user’s 3D virtual avatar face in real time using electrodes attached around the eyes. To implement this FER system without an extensive individual calibration process, a new concept named virtual blend shape weight (vBSW) was proposed, and a two-step FER approach consisting of classification and regression steps was employed. Although our FER system employed only ten electrodes attached to the VR HMD frame, online experiments with eleven participants demonstrated that it was possible to capture the user’s various lip and eyebrow motions continuously, which the conventional fEMG-based FER systems were not able to do. Additionally, to replicate more realistic avatar eye motions, we developed methods for real-time eye blink detection and eye gaze tracking using an electrooculogram (EOG) recorded using the same electrodes and incorporated them into the proposed fEMG-based FER system. Our proposed FER system allows the continuous tracking of facial expression changes by seamlessly adjusting the shapes of the lip, the eyebrows, and the eyes. To the best of our knowledge, this is the first study that implemented the continuous tracking of facial gestures with a limited electrode configuration in the VR HMD environment.

The remainder of the literature consists of the following sections: The Materials and Methods Section provides detailed information of the subjects, equipment, experimental protocols, signal preprocessing, and methods for FER. The Results Section presents the performance of our proposed system, both quantitatively and qualitatively. The Discussion and Conclusion Sections discuss some issues regarding our system and provide future prospects.

## 2. Materials and Methods

### 2.1. Subjects and Materials

**Figure 1:**
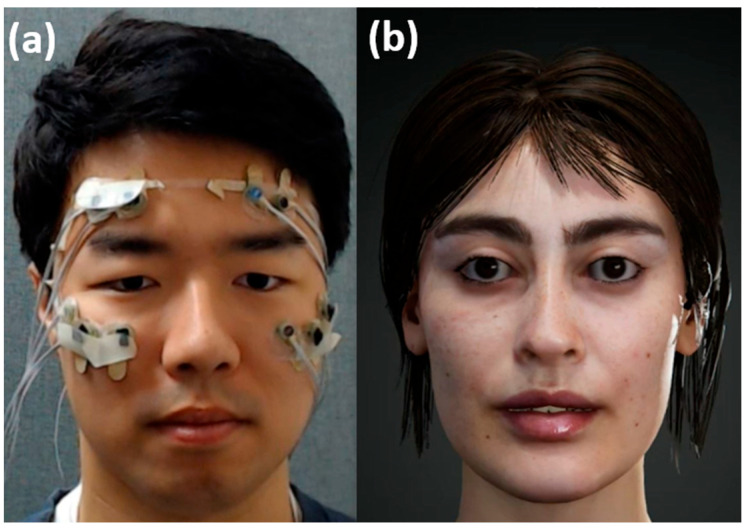
(**a**) A participant (third author of the paper) wearing an electrode-embedded plastic frame; (**b**) a 3D avatar used in the study.

Eleven healthy male participants (age: 28.36 ± 3.55) participated in this study. None of the participants reported any serious health problems, such as Bell’s palsy, stroke, or Parkinson’s disease, that might affect the study. Before conducting the experiments, all the participants were given a detailed explanation of the experimental protocols and signed a written consent form. The participants received monetary compensation for their participation in the experiments. The study protocol was approved by the Institutional Review Board (IRB) of Hanyang University, South Korea (No. HYUIRB-202209-024-1). A commercial biosignal acquisition system (ActiveTwo; BioSemi Inc, Amsterdam, The Netherlands) was used to record fEMG and EOG signals. Both signals were recorded at a sampling frequency of 2048 Hz. We attached ten active electrodes to plastic film as shown in Figure 1a. The thin plastic film was designed based on the shape of the pad of a commercial VR HMD (Samsung Gear VR 2019; Samsung Electronics, Seoul, Republic of Korea) to expose as much of the face area as possible. We employed the transparent plastic film instead of the actual VR HMD to quantitatively evaluate the FER accuracy by comparing the actual facial expressions with the expressions replicated on the avatar face. This experimental set-up was also used to determine some parameters relating the fEMG patterns with blend shape weights (BSWs) of the avatar face, for which three male participants (age: 29 ± 1.73) were enrolled. We implemented a 3D virtual avatar in the Unity environment, as shown in Figure 1b. Matlab ver. R2019a and R2015a (MathWorks, Natick, MA, USA) were used to process biosignals and predict the facial motions.

### 2.2. Acquisition of Calibration Data

**Figure 2:**
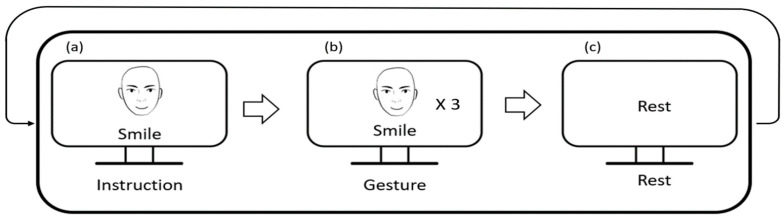
A schematic diagram of the experimental protocol. (**a**) Presentation of the next facial expression to the participant in a random order; (**b**) task performing step: the participant replicates the given facial expression repeatedly 3 times for 3 s; (**c**) a resting period lasting for 3 s.

All participants underwent a short calibration session to build an individual machine learning model. During the calibration session, they were asked to sit in front of a 24-inch LCD monitor that provided visual instructions. Each experimental trials for calibration consisted of three steps (see Figure 2). In the first step, the participants were informed about the next facial expression that they should make using both images and text. In the second step, the participants were asked to make the designated facial expression three times repeatedly for 3 s. In the last step, the participants were asked to relax their facial muscles and prepare for the next trial. The facial gestures employed in this study were selected from the facial action coding system (FACS) [20], which is a famous standard dataset widely used in facial expression recognition studies [17,21,22]. Among the tens of facial actions in the FACS, we picked eight of them considering the distance between the locations of facial muscles and the electrodes. It is to be noted that horizontal eye movement separated into left- and right-directional eye movements in the FACS, but we regarded it as a single facial action. Table 1 shows the full list of facial gestures used in our FER system.

### 2.3. Signal Preprocessing and Feature Extraction on Biosignals

Since fEMG and EOG signals could be measured using surface electrodes, both signals were recorded using the same electrodes. fEMG signals were acquired from all ten electrodes, while horizontal EOG (hEOG) signals were extracted from four electrodes (the two rightmost electrodes and two leftmost electrodes; see Figure 1a).

The signal processing steps of the fEMG signals are as follows. The acquired fEMG signals were notch filtered at 58–62 Hz to eliminate AC power noises, followed by a fourth-order Butterworth bandpass filter with a bandwidth of 20–450 Hz. The filtered fEMG signals were segmented using a 100 ms sliding window with a 50 ms overlap. The sliding window started at 0 ms and lasted until the end of the signal duration with fixed intervals of 50 ms, resulting in an estimate of the facial expression every 50 ms. Following this, we computed the root-mean-squared (RMS) values for each segment using

(1) $$xRMS=\sqrt{\frac{1}{N}\sum_{n=1}^{N}{\left|Xn\right|}^{2}},$$

where *Xn* represents the *n*-th sample value and *N* represents the number of samples in the sliding window. The RMS values were then smoothed by applying a fourth-order Butterworth lowpass filter with a 0.05 Hz cutoff for each trial.

The EOG signals were processed in the following steps. As shown in Figure 1a, there were no electrodes on the left and right sides of the eyes. Therefore, EOG signals from the two leftmost electrodes were averaged to estimate the left electrode hEOG value. Similarly, EOG signals on the two rightmost electrodes were averaged to estimate the right electrode hEOG value. In same way, the EOG values from four electrodes above the eyes and the EOG values from six electrodes below the eyes were averaged to estimate the vertical EOG (vEOG) values, which were then used to identify eye blinks.

### 2.4. Acquisition of Facial Motion Data to Relate BSW and fEMG

To relate the facial motions to the fEMG signals, additional facial motion data were collected with a webcam using a camera-based facial motion capture software package (f-clone; https://vimeo.com/219844273, accessed on 5 February 2022) from three participants. More specifically, we collected BSWs consisting of 29 different categories that represent various facial motions, such as ones with the left corner of the mouth moved up, the mouth centered, the mouth open, and the right cheek raised [23]. The list of all 29 BSWs is provided in Supplementary Materials Table S1. We used the raw BSWs directly without any preprocessing. Among the 29 BSWs, 9 BSWs were selected because they are closely related to the facial expressions that we were trying to capture. Table 2 shows the selected BSWs for each facial expression. Note that the lip motions were affected by multiple BSWs, while the eyebrow motions of the left and right eyebrows were affected by only one BSW. The collected BSWs data and simultaneously recorded fEMG signals were then used to formulate the relationship between BSWs and fEMG, which are described in Section 2.5.

### 2.5. Virtual Blend Shape Weights

To predict and reconstruct facial motions from fEMG signals without actual BSWs acquired from each user, we proposed a new concept called ‘virtual blend shape weight (vBSW)’. The vBSW is an artificially generated BSW from each user’s fEMG signal data, which has a unique weight combination of BSWs determined by analyzing simultaneously acquired BSWs and fEMG signals in the preliminary experiment with three participants. The vBSWs were calculated by multiplying weights of each facial expression with the averaged preprocessed fEMG signal. The weight is a value assigned to each BSW, which ranges from 0 to 1. Each facial expression has its own unique combination of weights, so that the combination of weights for a specific facial expression represents how much each BSW value changes when the facial expression is generated. The weight combination of each facial expression was empirically determined based on the actual BSWs captured using the f-clone program.

### 2.6. Prediction of BSWs from Facial Biosignals

We implemented a two-step prediction procedure that consisted of classification and regression. The first step classified five discrete lip motions and two eyebrow motions. After the classification step, the second step conducted linear regression prediction of continuous facial motion based on fEMG signal amplitudes.

(1) Classification Step
  The Riemannian manifold-based pattern classification method, which is identical to that in our previous study [19], was implemented. For each preprocessed fEMG signal, a C × C sample covariance matrix (SCM) was computed as C_w_ = 1/(S − 1)x_w_ x_w_^T^, where x_w_ is a segmented fEMG signal of which the dimension is W × C, w = 1, 2, 3,…, W. Here, W is the number of windows in the segment, S is the number of samples in a single segment, and C is the number of channels. As described in the previous study, the space of SCM can become a Riemannian manifold, and by mapping the SCM onto a tangent space, the Riemannian manifold-based fEMG feature can be computed. Specifically, the SCM of the segmented fEMG signal was computed and mapped onto the tangent space formed by a reference SCM. The method for computing the reference SCM is described in [24]. Then, the extracted features were used to train a linear discriminant analysis (LDA) classifier. The fEMG data recorded during the calibration sessions were used to train the classifier model. After building the classifier model, the 100 ms fEMG sliding window was fed into the model during the online session, resulting in a classification result at every 50 ms. Here, LDA classification models are made for each lip motion detected and brow motion detected, respectively, making it possible to track the lip and brow motions independently.
(2) Regression Step
  After the classification of discrete facial motions, a linear regression model-based support vector machine (rSVM) was used to predict the continuous facial motion. As mentioned in Section 2.5, each facial expression category had its own combination of vBSW weights. Individual rSVM-based prediction models were created for each facial expression of each user using calibration fEMG signals and the vBSW of each facial expression. As a result, once the two-step model was trained with the calibration fEMG data, in the online experiments, the model first classified the facial expression from the given fEMG signals, and then it predicted the intensity of the facial expression in the form of a combination of BSWs. Again, according to the separated LDA models of the lips and brows, regressions of the lips and brows were achieved independently.

**Figure 3:**
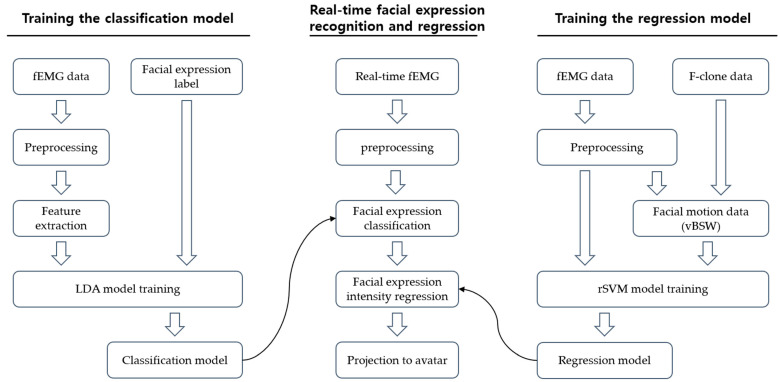
Overall procedure of the facial expression prediction system. Left panel presents the sequence of classification model training. Right panel presents the sequence of regression model training. Middle panel presents the process for real-time facial expression recognition and projection of the predicted facial expression and its intensity onto the avatar face.

Flowcharts for the classification and regression steps and the procedure of the real-time FER system are depicted in Figure 3.

### 2.7. Eyeball Movements and Eye Blink Detection

**Figure 4:**
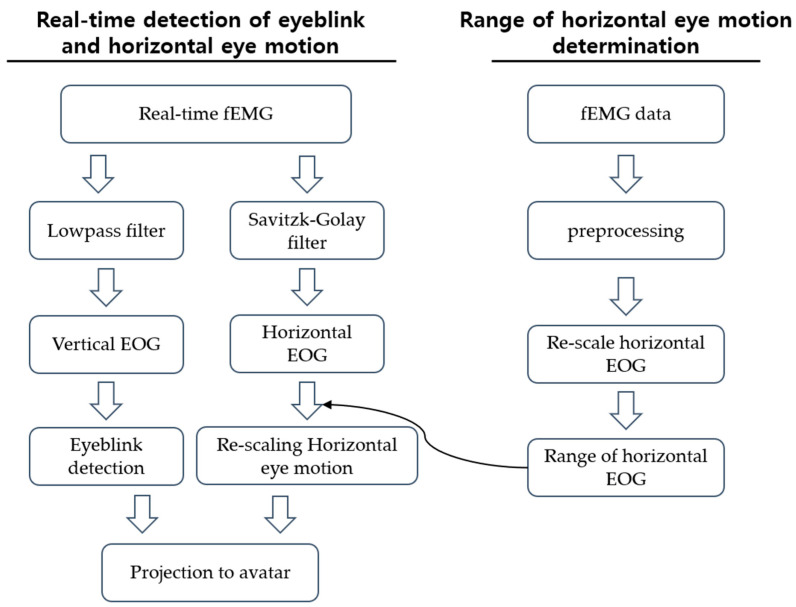
The right panel shows the overall procedure of eye blink and horizontal eye motion detection. The left panel presents the detailed method determining minimum and maximum ranges of horizontal EOG for re-scaling of the horizontal eye motion.

For eyeball movement tracking, a simple EOG-based eye tracking method was employed. Traditionally, EOG electrodes are located at the leftmost and rightmost sides of the eyes and above and below each eye [25]. Then, horizontal and vertical components of the EOG signals are used to determine the eye movement directions [26]. In this research, as mentioned above, we calculated hEOG as the difference between the average values of the two leftmost electrodes and the two rightmost electrodes. The hEOG value was then used to estimate the horizontal eyeball movements. Since the BSWs of horizontal eyeball movement ranges from −1 to 1, each representing the rightmost direction and the leftmost direction, the range of hEOG was rescaled between −1 and 1 by mapping the minimum and maximum values of hEOG between −1 and 1, respectively. In general, there is a drift in the EOG signal baseline, which hinders the steady tracking of eyeball directions [25,27]. To overcome this issue, we applied a continuously updating centerline method that calculated the average hEOG for the most recent 10 s and considered it as the EOG signal acquired when the eyes were located at the center. Please note that only horizontal eyeball tracing was implemented in our system. Eye blink detected was based on an algorithm called the summation of the first-order derivative within a window [28]. For the detection of eye blinks, vEOG was used, which was calculated by subtracting the mean value of electrodes below the eyes from the mean value of electrodes above the eyes. A schematic diagram of real-time eye blinks and eye motion detection is presented in Figure 4.

### 2.8. Real-Time Avatar Interaction

**Figure 5:**
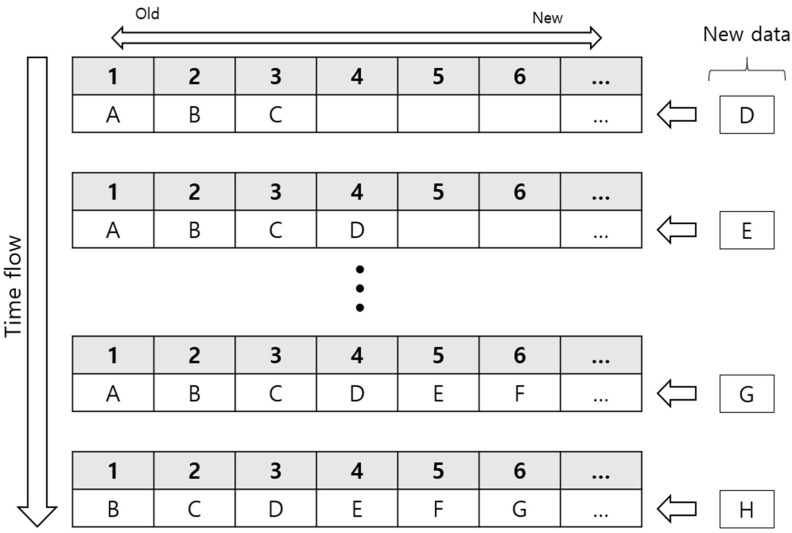
FIFO-based queue for tracking N-most recent data.

The two-step facial motion prediction model, an eye blink detection model, and an eye movement tracking model were incorporated into a Matlab-based real-time facial motion capture system and projected onto a Unity-based 3D avatar face in real time. The real-time program acquires the fEMG signals from a commercial biosignal acquisition system every 50 ms. fEMG signals were preprocessed and stacked in the queue until the length of the stacked data reached 100. After 100 samples were stacked, the system initiated the two-stage prediction model for facial expression prediction. After starting the prediction sequence, the window of fEMG data was repeatedly updated every 50 ms by newly acquired fEMG data. This updating process was conducted by a first-in first-out (FIFO) paradigm, as depicted in Figure 5.

As shown in Figure 5, adding new fEMG data at the end of the queue and removing the oldest fEMG data from the front of the queue were performed simultaneously so that the queue always contained the latest 100 samples, which occurred every 50 ms. Each time the queue was renewed, the two-step prediction model analyzed new data in the queue to classify the facial expression and estimate the intensity of the expression in order to generate the updated combination of BSWs. BSWs from the two-step prediction model were then combined with other BSWs representing eye movements and eye blinks. All the BSWs were subsequently sent to the avatar module.

### 2.9. Evaluation Methods

To quantitatively evaluate the performance of the proposed real-time facial motion capture system, the participants were asked to make various facial expressions after the completion of the individual calibration session. The facial expressions replicated on the avatar face were presented to the participants in real time, and no participant reported any feeling of delayed visualization of their facial expressions. The user’s facial expressions could be displayed without a delay because the generation of fEMG generally precedes the actual muscular movements. In the online experiments, five lip motions, two eyebrow motions, and three eye motions were tracked. In the experiments, the lip and eyebrow motions were repeated three times, while the horizontal eyeball movements (shifting eye gaze to the left and right) were repeated five times in a separate session. While the participants were performing the given tasks, videos of the participants’ facial motion were taken along with the biosignals. The facial video data taken from eleven test participants during online experiments were used to quantitatively evaluate the performance of our FER system, as follows: To evaluate the performance of our system, the predicted facial motions on the avatar were quantified, and the values were compared to the actual facial motions taken from video images, which were also quantified. The quantification of the facial motion was represented by four features for each lip motion and two features for each eyebrow motion. The features were calculated from factors of facial motions such as the length of the lip, the length between the upper and lower lip, the positions of the corners of the lip, and the height of the eyebrow. The features were evaluated based on the following equation.

*x* = (FFV_emotion_ − FFV_neutral_)/FSV (2)

where FFV is face factor value and FSV is face size value

In the equation, the computed value of *x* represents each of the features, acquired by dividing the differences between facial factor values of the neutral face and the other facial expressions by the face size value, which compensates for the difference in sizes between the real face and avatar face. The face factor value (FFV) represents a variable value of facial motions mentioned above. For example, the length of the lip, one of the FFVs, would be different on a neutral face and s smiling face. In addition, the length of the lip would be different for each different intensity of smiling face; therefore, FFV allows researchers to quantify the continuous changes in real and avatar faces. By subtracting the FFV_neutral_ from FFV_emotion_, the difference in the facial motion factor values between a certain facial expression and a neutral face can be computed. Face size value (FSV) represents the face size factors, which are the horizontal length of the face, calculated by the distance between ear to ear of the face and the vertical length of the face, calculated by the distance between the glabella to the bottom of the chin. Since the size of the avatar face and the real face are different, the FSV was employed to normalize the feature. For five facial expressions, except the neutral face, the features were calculated for each real face and avatar face. Then, the Pearson correlation between real and avatar faces was calculated. Eyeball movement and wink detection were also counted, and the accuracy of eyeball movement detection was evaluated by counting binary true and false classifications for each left and right movement.

## 3. Results

### 3.1. Comparison between Real Face and Predicted Face

**Figure 6:**
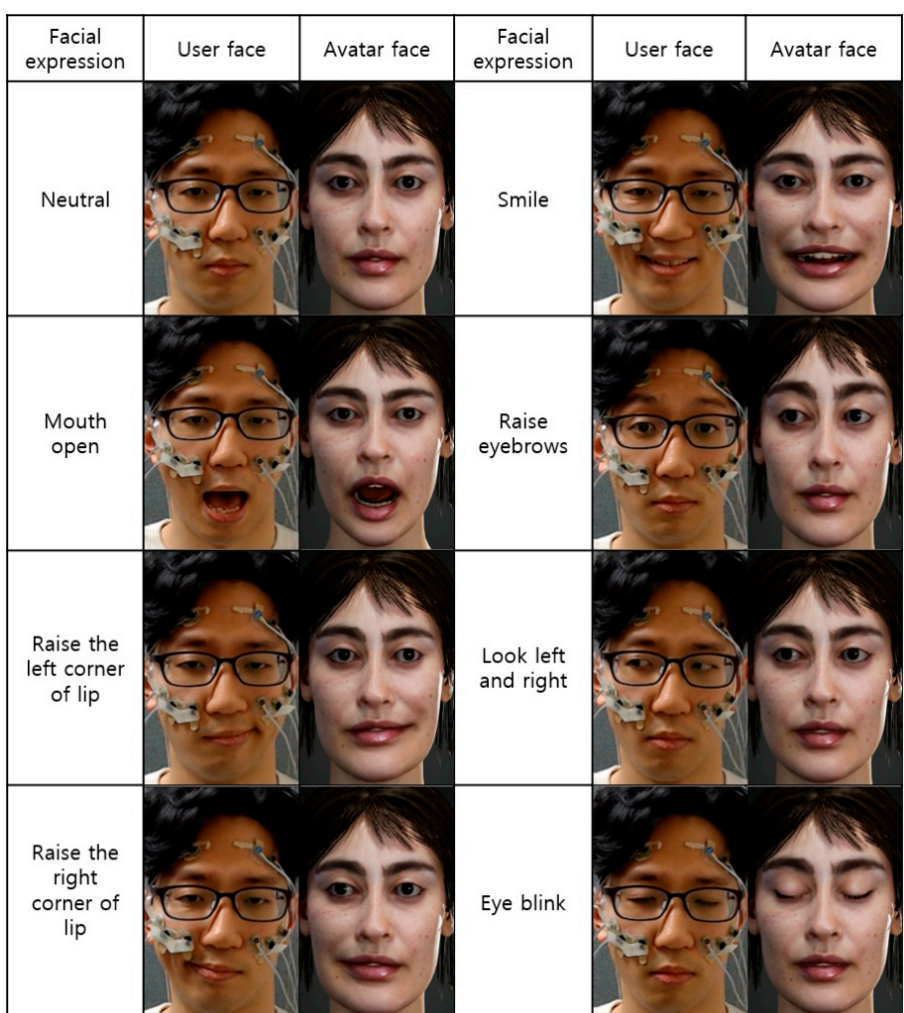
Comparison between actual face and predicted avatar face.

All eleven participants repeated each facial motion (five lip motions, two eyebrow motions, two eye motions, and eye blinks) three times in the online experiments. Figure 6 compares the real face and the avatar face reconstructed in real-time with respect to various facial expressions (note that the person in the figure is the first author of this article). As shown in the figure, the avatar face was able to successfully mimic six different lip and eyebrow motions, track horizontal movements of the eyeball, and detect eye blinks. The demonstration video can be found on YouTube^TM^ (https://youtu.be/aIg6u2_XDmw, accessed on 12 February 2023), where real-time testing with a participant is shown.

### 3.2. Quantitative Evaluation of Facial Motion Estimation

The performance of the developed facial motion capture system was evaluated after the online experiments were finished. As mentioned in the Methods Section, the Pearson correlation between the features of the real face and avatar face was calculated, as presented in Table 3. The average Pearson correlation was 0.88, and most of the participants exhibited correlation values larger than 0.85. Table 3 also shows the accuracy of estimating eye blinks and eye motion directions. The accuracy, precision, recall, and F1 score were calculated from the confusion matrix of each of the eye blinks and eye motion trials. Among these four values, a false negative refers to the absence of an action in both the real and avatar faces. However, in continuous performance testing, it is impossible to count the absence of eye blinks and eye motions in discrete numbers. Therefore, the false negative values were fixed at 0 for both eye blink and eye movement detection. The F1 scores of eye blink detection and eye motion detection were reported to be 83.3% and 91.5%, respectively.

As shown in the table, the recall was relatively higher than the precision was in eye blink detection, implying that our system captured the eye blinks fairly well, but it also overreacted even without actual eye blinks or eye motions. Most of the false positive detections of eye blinks were caused by eyebrow motions. Since eyebrow motions also elicit fEMG signals similar to vEOG signals by eye blinks, fast eyebrow motions could be misclassified as eye blinks.

## 4. Discussion

Our previous study simply classified discrete facial expression in a VR HMD environment [19]; however, in this study, we further investigated the possibility of enabling the continuous and more natural tracking of facial motions by employing an SVM-based linear regression method, which is referred to as a two-step prediction procedure in this paper. It is to be noted that the ultimate goal of facial recognition technology would be to exactly replicate arbitrary facial expressions without any classification steps; however, as the muscles predominantly related to lip and jaw motions are located in the lower part of the face, which is far from the surface electrodes embedded in the VR-HMD, it was highly difficult to trace the arbitrary facial motions in real time with high estimation accuracy. By limiting the number of recognizable facial expressions, it was possible to achieve the stable and fast real-time prediction of facial motions. More specifically, our previous work achieved 85% accuracy with 11 facial expressions [19]. However, it is to be noted that the purpose of the present study was to track continuous changes in facial expressions and project them onto the avatar face in real time. To this aim, every single recognition process at every 50 ms should be highly accurate. Otherwise, the avatar face will be disrupted or distorted due to the suddenly appearing single wrong prediction result. Therefore, 85% accuracy seemed to be quite low to realize naturalistic facial tracking. Since our current study reduced the number of recognizable facial expressions to six, the classification accuracy reached 96.65%, which was 11.65% higher than that in our previous study. With this improved accuracy achieved by sacrificing the number of recognizable facial expressions, our system could demonstrate the stable and robust performance of real-time facial motion capture such as that shown in the demonstration video. Nevertheless, we still believe that the classification accuracy can be further improved by developing new algorithms in future studies, thereby allowing the increment of the number of facial expressions classifiable in our FER system.

In the regression step of the two-step prediction procedure, vBSWs were created and used instead of the actual BSWs recorded with fEMG data. The actual BSWs collected in this research were recorded with a webcam, and thus, they cannot be directly applied to VR users whose eyes are covered with a VR-HMD. By assuming that the intensity of facial expression has a linear relationship with the amplitude of the fEMG signal, we multiplied smoothed fEMG signals by a unique combination of weights to generates the vBSWs. The weights represent the contribution of each BSWs to certain facial expressions, and the weights of each facial expression were determined by empirical analysis of the actual BSWs recorded for three participants. Not only facial expressions but also eye gaze is an important factor that reflect human emotion and intention, as people can sometimes recognize others’ intention through the eye gaze direction. This importance also applies in virtual environments. Research has shown that intentionally controlled eye gaze and eye blinks of an avatar provide high-quality avatar realism and help people become more immersed in the virtual environment [12]. In this regard, the present study employed eyeball tracking and eye blink detection to realize more realistic avatar face reconstruction.

There are some limitations that need to be addressed. Although the developed system classifies various facial expressions, some important facial motions that express a user’s emotion, such as the ‘oh’ face and the ‘upset’ face, were not included in the current expression list. Additionally, the eyeball movement tracking only traces horizontal movements due to the difficulty of distinguishing vertical eyeball movements and eye blinks. A future study will be conducted on these excluded facial expressions. For example, there are several studies that aim to eliminate eye blink artifacts from all-direction EOG [27]. Additionally, the commonly applied EOG baseline removal algorithm was implemented simply by averaging hEOG value in the most recent 10 s, but there are potentially better algorithms [29,30] that might be applied in future studies. In addition to these limitations, there can be some threats to validity, which might be a potential drawback in practical applications. One example is the test–retest reliability issue: whenever the user of this system re-wears the device, the locations of electrodes on the face would be slightly shifted, which might lead to degradation of the overall performance. The employment of domain adaptation strategy may be a potential solution to address this issue [15]. Another possible issue might be the limited processing power of mobile edge devices, which might hinder the real-time processing of fEMG and realistic avatar visualization. Simplification of the realistic avatars seems to be the only available solution at the current level of technology [31]; however, as the processing power of mobile edge devices is rapidly increasing, it is expected that this issue can be overcome in the near future.

Although several studies attempted to recognize facial expressions without a camera in a VR environment, only a few of them achieved the high-accuracy classification of facial expressions in real time [32]. To the best of our knowledge, there is no system that replicates facial expressions, eye blinks, and eyeball movements in an all-in-one platform using the same electrodes. In addition, our research is the first one to achieve naturalistic facial regression prediction among the studies based on fEMG approach with limited electrode locations. In summary, the system developed in this study is superior in many ways, including its low cost, light weight, and capacity of tracking facial expressions and eye movements at the same time with the same set of electrodes. In view of this, we believe that our study is the first step towards a practical EMG-based facial tracking system that can be commercialized in the near future. As seen in the Results Section, the system successfully regenerated whole facial motions in real time, and future studies will allow us to expand the boundaries of classifiable facial expressions and make the system capable of tracking every possible facial expression that a human can make. We believe that the future system will be a powerful tool for representing users in the virtual reality environment and will be a valuable and competitive technology in the VR-SNS field.

## 5. Conclusions

In this study, we implemented a machine-learning-based real-time facial motion prediction system that can trace various facial motions of VR users and project them onto a 3D avatar face in real time. Our system does not require any extra cameras to recognize the facial motion, and it can be realized with only ten surface electrodes for the precise prediction of five lip motions, two eyebrow motions, horizontal eyeball movements, and eye blinks. As a result, the re-enacted facial expressions of an avatar showed high similarity when they were compared to those of the real face, with the mean Pearson correlation value of 0.88. As also seen in the demonstration video, the system showed very stable reconstructions of facial expressions.

## Figures and Tables

**Table 1:** List of predictable facial expressions.

Category	Facial Expressions
Lip motions (5)	Neutral; mouth open; raise the left/right corner of the lip; smile.
Eyebrow motions (2)	Neutral; raise the eyebrows.
Eye motions (2)	Eye blink; horizontal movement of eyeballs.

**Table 2:** Facial expressions and related BSWs. Numbers in the parenthesis represent the number of BSWs related to each facial expression.

Facial Expressions	Related BSWs
Mouth open (3)	Mouth open; mouth left/right spread.
Smile (5)	Mouth open; mouth left/right spread; cheek left/right up.
Raise the left/right corner of the lip (2)	Mouth left/right spread; cheek left/right up.
Raise the eyebrows (1)	Brow left/right up.

**Table 3:** Correlation between real and avatar facial motions and accuracy, precision, recall, and F1 score of eye blinks and eye motions.

	Facial Motion	Eye Blink				Eye Motion			
	Correlations	Accuracy (%)	Precision (%)	Recall (%)	F1 Score	Accuracy (%)	Precision (%)	Recall (%)	F1 Score
Subject 1	0.85	69	90.9	74.1	0.816	100	100	100	1
Subject 2	0.854	100	100	100	1	100	100	100	1
Subject 3	0.794	81	81	100	0.895	83.3	83.3	100	0.909
Subject 4	0.826	100	100	100	1	100	100	100	1
Subject 5	0.888	50	55	84.6	0.667	100	100	100	1
Subject 6	0.859	80	92.3	85.7	0.889	50	50	100	0.667
Subject 7	0.929	80	80	100	0.889	100	100	100	1
Subject 8	0.863	94.3	100	94.3	0.971	71.4	71.4	100	0.833
Subject 9	0.973	58.8	0.58.8	100	0.741	100	100	100	1
Subject 10	0.871	28.6	28.6	100	0.444	75	100	75	0.857
Subject 11	0.977	73.9	89.5	81	0.85	66.7	100	66.7	0.8
Average	0.88	74.1	79.6	92.7	0.833	86	91.3	94.7	0.915

## Data Availability

The data presented in this study are available on request from the corresponding author.

## Supplementary Materials

The following supporting information can be downloaded at: https://www.mdpi.com/article/10.3390/s23073580/s1, Table S1: List of all blendshape weights from f-clone system; Table S2: List of all facial gestures reconstructed in this study and blend shape weights related to each facial expression; Table S3: Detailed structure of fEMG dataset for calibration.
