# Supplementary material for: Facial Motion Capture System Based on Facial Electromyogram and Electrooculogram for Immersive Social Virtual Reality Applications
Source: Sensors (Basel). 2023 Mar 29;23(7):3580. doi: 10.3390/s23073580 (PMC10099104; doi:10.3390/s23073580)
Supplement: Supplementary file 1 [file sensors-23-03580-s001.zip › sensors-2186968-supplementary.pdf]

Supplementary Table S1. List of all blendshape weights from f-clone system

# 1~15	Blend Shape Weights	#16~29	Blend Shape Weights
1	Head rotation X	16	Mouth Left Spread
2	Head rotation Y	17	Mouth Right Spread
3	Head rotation Z	18	Mouth Left Frow
4	Brow Left Up	19	Mouth Right Frow
5	Brow Left Down	20	Mouth Left Centerize
6	Brow Right UP	21	Mouth Right Centerize
7	Brow Right Down	22	Cheek left Up
8	Brow Centerize	23	Cheek Right Up
9	Left Brow Outer Down	24	Left Eye rotation X
10	Right Brow Outer Down	25	Left Eye Rotation Y
11	Eye close Left	26	Left Eye Rotation Z
12	Eye close Right	27	Right Eye rotation X
13	Mouth Open	28	Right Eye rotation Y
14	Mouth Left Smile	29	Right Eye rotation Z
15	Mouth Right Smile		

Supplementary Table S2. List of all facial gestures reconstructed in this study and blend shape weights related to each facial expression.

# 1~8	Facial expressions	BSW and v-BSW
1	Neutral	-
2	Mouth open	Mouth open, Mouth left spread, Mouth right spread
3	Smile	Mouth open, Mouth left spread, Mouth right spread, Cheek left up, Cheek right up
4	Raise the left corner of the lip	Mouth left spread, Cheek left up
5	Raise the right corner of the lip	Mouth right spread, Cheek right up
6	Raise the eyebrows	Brow left up, Brow right up
7	Eye blink	Eye lid close
8	Horizontal eye motion	Right eye rotation X, Left eye rotation X

Supplementary Table S3. Detailed structure of fEMG dataset for calibration. Note that the eye-blink detection does not require training.

Details of fEMG dataset	
# of trial per facial expression	1
Length of single trial	3 sec
Length of each signal window	100 ms
Length of overlap between windows	50 ms
# of signal windows per trial	59
# of channels	10
# of facial expressions	7 (except eye blink)
